# Elevated Allele Frequency of a Common Germline *PDCD1* Variant Associated with Leukocytosis and Thrombocytopenia in Acute Myeloid Leukemia Treated with HDCT/ASCT

**DOI:** 10.3390/cancers18213537

**Published:** 2026-11-03

**Authors:** Katja Seipel, Ranja Leuenberger, Henning Nilius, Michele Hoffmann, Martina Bertschinger, Marie-Noelle Kronig, Ulrike Bacher, Thomas Pabst

**Affiliations:** 1 Department of Medical Oncology, Inselspital, Bern University Hospital, University of Bern, 3010 Bern, Switzerland; ranja.leuenberger@students.unibe.ch (R.L.); michele.hoffmann@insel.ch (M.H.); martina.bertschinger@insel.ch (M.B.); marienoelle.kronig@insel.ch (M.-N.K.); 2 Department for Biomedical Research, University of Bern, 3008 Bern, Switzerland; 3 Department of Clinical Chemistry, Inselspital, Bern University Hospital, University of Bern, 3010 Bern, Switzerland; henning.nilius@insel.ch; 4 Department of Hematology, University Hospital Bern, 3010 Bern, Switzerland; veraulrike.bacher@insel.ch

**Keywords:** programmed cell death protein 1 (PD-1), single nucleotide polymorphism (SNP), minor allele frequency (MAF), acute myeloid leukemia (AML), genetic risk

## Abstract

Background: Germline variants of immune-regulatory genes may affect immune checkpoint functions, disease susceptibility, disease features, and treatment response in acute myeloid leukemia. Methods: We analyzed the prevalence and potential impact of the *PDCD1* gene variant rs6710479 in AML patients eligible for high-dose chemotherapy and autologous hematopoietic stem cell transplantation as consolidation treatment in first complete remission. Results: The *PDCD1* rs6710479 was prevalent at elevated minor allele frequencies (MAF 0.52) in AML patients, compared to the allele frequencies reported for the European population (MAF 0.43). Baseline blood count profiles differed across *PDCD1* genotypes, suggesting a link between *PDCD1* rs6710479 and disease-associated levels of thrombocytopenia and leukocytosis. Conclusion: The germline *PDCD1* rs6710479 variant may associate with AML disease risk and specific hematological disease features.

## Simple Summary

Programmed cell death protein 1 (PD-1) is an immune checkpoint receptor on the surface of T-cells. This is the first report on the prevalence and impact of germline *PDCD1* variants in AML. The *PDCD1* gene variant rs6710479 was prevalent at elevated allele frequencies in AML patients eligible for high-dose chemotherapy and autologous hematopoietic stem cell transplantation. Baseline clinical characteristics of AML patients varied according to *PDCD1* genotype, indicating an association of the *PDCD1* rs6710479 variant with leukocyte and platelet imbalance in AML disease.

## 1. Introduction

High-dose chemotherapy and autologous stem cell transplantation (HDCT/ASCT) is an effective consolidation treatment for eligible patients with favorable to intermediate risk acute myeloid leukemia (AML) who achieve minimal residual disease (MRD) negativity in first remission after induction chemotherapy [1]. In this patient setting, HDCT/ASCT offers better relapse-free and overall survival (PFS and OS) than chemotherapy consolidation alone. Nonetheless, considerable variability in outcomes following HSCT/ASCT remains, highlighting the necessity for additional prognostic indicators to improve therapeutic decisions.

There is growing evidence that the development of cytotoxic T cell dysfunction, particularly senescence and exhaustion, plays a role in the development of AML and other hematological malignancies [2,3,4]. Effector T-cell exhaustion and the growth of regulatory T cells within the bone marrow microenvironment can compromise antitumor immunity [5]. A number of immune checkpoint molecules are essential for controlling these immune interactions, including lymphocyte activation gene 3 (LAG3, CD223), cytotoxic T-lymphocyte-associated protein 4 (CTLA-4, CD152), and programmed cell death-1 (PD-1, CD279) [6]. Among these, PD-1 and LAG3 have emerged as critical drivers of T-cell exhaustion and promising targets for next-generation tumor immunotherapies [7,8]. Elevated PD-1 expression in the bone marrow correlates with poor prognosis, T-cell exhaustion, and resistance to standard therapies [9]. Elevated PD-1 expression on circulating T cells correlates with inferior outcome after HDCT/ASCT [10].

The *PDCD1* gene, located on chromosome 2, encodes the receptor protein PD-1 expressed on activated CD4+ and CD8+ T cells, natural killer cells, and dendritic cells. Upon binding to ligands PD-L1/PD-L2, the activated PD-1 delivers inhibitory signals that suppress T-cell proliferation and cytokine production. Blocking PD-1 can restore T-cell cytotoxic activity and enhance anti-tumor responses, particularly when combined with LAG-3 inhibition [4].

The functions of immune checkpoints can vary based on the genetic variants of the respective genes [11]. We previously analyzed common polymorphisms in immune-regulatory genes *CTLA4*, *LAG3*, and *PDCD1* in hematological malignancies and reported allele frequencies, associated disease risks, associated baseline characteristics, and associated clinical outcomes [12,13,14,15,16].

This retrospective study concentrated on assessing the risk linked to the common *PDCD1* variant rs6710479 in relation to AML pathogenesis, possible effects on disease characteristics, and potential impact on clinical outcomes following HDCT/ASCT. The aim of the study was to elucidate how *PDCD1* genetic variability influences AML susceptibility and treatment outcomes by genotyping a well-defined patient cohort and examining the relationship between the genetic variant and clinical parameters. The identification of risk-related gene variants may improve the understanding of disease mechanisms and aid in tailoring immunotherapy approaches for AML patients.

## 2. Materials and Methods

### 2.1. Gene Sequence and Gene Risk Analysis

Genomic DNA was obtained from mononuclear cells (PBMCs) isolated from the peripheral blood of AML patients in complete remission (CR) following induction therapy. *PDCD1* gene intron one was PCR amplified and submitted to Sanger sequencing as described [12]. Sanger sequencing results clearly indicated homozygosity of the SNP (allele dosage corresponding to signal strength 2:0 or 0:2) or heterozygosity (allele dosage corresponding to signal strength 1:1). Minor allele frequencies and gene risk were calculated on GeneRiskCalc (version 1.0), a web-based tool for genetic risk association analysis in case–control studies (https://sites.google.com/view/generiskcalc/home/gene-association-calculator) [17].

### 2.2. Study Design and Patient Cohort

We carried out a retrospective single-center analysis in a cohort of 139 patients (pts) diagnosed with AML between 2006 and 2023 at Inselspital, University Hospital Bern, Switzerland. The cohort selection criteria were consecutive favorable- and intermediate-risk AML in first MRD-negative CR after intensive induction treatment and eligible for ASCT consolidation. MRD was evaluated with quantitative PCR for NPM1mut, inv(16), or t(8;21) [18]. Of the 203 pts eligible for ASCT consolidation, 167 pts signed the general consent, 146 pts had a sufficient number of SC collected, and 139 pts had complete clinical data. The AML classification at diagnosis was assessed according to the French–American–British (FAB) system [19]. Patients were categorized into prognostic groups according to the molecular genetics and karyotyping of their AML cells, following the guidelines set forth by the European Leukemia Network (ELN) as detailed [18,20]. The AML patients underwent two cycles of induction therapy based on the conventional “7 + 3” regimen, comprising cytarabine and an anthracycline. Anthracyclines were omitted in cycle 2 for patients above 60 years. Patients achieving complete remission after cycle 2 were admitted to receive consolidation treatment with autologous stem cell transplantation (ASCT). Stem cells were harvested following recovery after cycle 2—or, alternatively, using vinorelbine mobilization [21]. The conditioning regimen was high-dose chemotherapy (HDCT) with melphalan and treosulfan followed by ASCT [22]. No maintenance treatment was given after ASCT. This research followed the guidelines of the Declaration of Helsinki and obtained approval from the Ethics Commission of the Canton of Bern (approval number 2025-00853, date of approval 24 April 2025). Consent was acquired from all participants. Clinical data were examined as previously described [13]. The reporting on the study cohort adhered to the STROBE statement (Strengthening the Reporting of Observational Studies in Epidemiology) [23].

### 2.3. Statistical Analysis

Three genotype-associated groups were identified based on the *PDCD1* gene polymorphism rs6710479: heterozygous carriers (CT), homozygous carriers of the minor allele (TT), and homozygous carriers of the major allele (CC). Complementary information on treatment procedures was obtained from the Marcell software Version 8.3.3.0, while clinical data was gathered from the EPIC electronic health record system at Bern University Hospital. When appropriate, the Chi-square test or Fisher’s exact test was used to compare categorical variables. The Kruskal–Wallis test was used to compare continuous non-parametric variables between groups, and the Mann–Whitney U test was used for pairwise comparisons. The Kaplan–Meier method was used to estimate progression-free survival (PFS) and overall survival (OS), which were then compared between groups using the log-rank test. The Cox proportional hazards model was used in both univariate and multivariable analyzes to find independent prognostic variables for OS and PFS. We evaluated the relationships between *PDCD1*, *CTLA4*, and *LAG3* common genetic variants with PFS and OS using univariable and multivariable Cox proportional hazards models. Multivariable models were adjusted for age (≤65 vs. >65 years), sex, and ELN risk category (adverse vs. not adverse). These models also included leukocytosis (WBC < 10 vs. >10 g/L), thrombocytopenia (PLT < 50 vs. >50 g/L), and the percentage of peripheral blood blasts at diagnosis (<50% vs. ≥50%). We calculated hazard ratios with corresponding 95% confidence intervals. Statistical analyses were performed with GraphPad Prism (version 11.0.0; GraphPad Software, San Diego, CA, USA) and RStudio (version 2025.05.1 + 513; Posit PBC, Boston, MA, USA) for conducting Cox regression analyses. A two-sided *p*-value of <0.05 was deemed statistically significant.

## 3. Results

### 3.1. Characteristics of the PD1 rs6710479 Variants

The single nucleotide polymorphism (SNP) rs6710479 is a common *PDCD1* germline variant with a minor allele frequency (MAF) estimated at 0.424 (gnomAD, samples size 78′496, access July 2026) and 0.434 (ALFA, sample size 22′086, access July 2026) in the European population, indicating a 32% prevalence of CC homozygotes, 49% CT heterozygotes, and 19% TT homozygotes. rs6710479 is an intronic SNP with a putative MZF1 transcription factor binding site in the minor allele [12].

### 3.2. Elevated Prevalence of PDCD1 rs6710479 in AML Patients

The sequences of intron one of the *PDCD1* gene were analyzed in peripheral blood cells of 139 AML patients who attained complete remission after induction therapy. Among these, 33 patients (24%) were found to carry two major alleles (CC), 68 patients (49%) carried one allele rs6710479 (CT), and 38 patients (27%) carried two alleles rs6710479 (TT).

**Figure 1:**
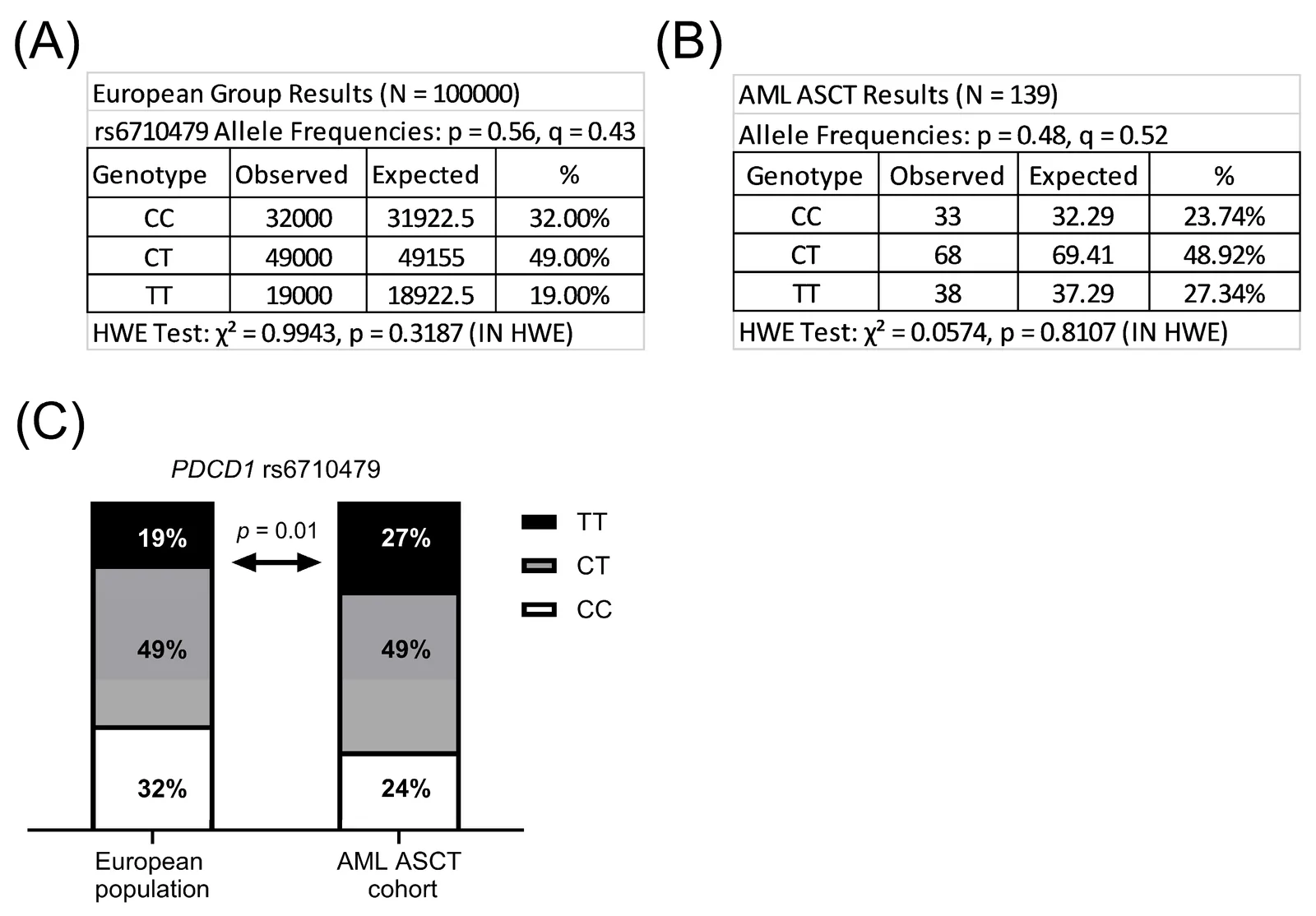
Prevalence of *PDCD1* rs6710479 in the local AML cohort and the broader European population. Allele frequencies of *PDCD1 rs6710479* in the European population (**A**) and within the local AML cohort (**B**). A comparative prevalence assessment of *PDCD1 rs6710479* between the local AML cohort and the European population (**C**). Hardy–Weinberg equilibrium (HWE) is referenced for genetic balance, with “p” denoting the major allele and “q” representing the minor allele.

The AML cohort’s allele frequencies were computed and contrasted with those of the European population (combined ALFA and gnomAD sample size 100′000). The minor allele frequencies differed, with MAF 0.52 in the AML cohort and MAF 0.43 in the European population (Figure 1A,B). The AML cohort had higher rs6710479 frequencies than the European control group, with 27% against 19% TT and 32% versus 24% CC, *p* = 0.01 (Figure 1C). A violation of Hardy–Weinberg equilibrium (HWE) may jeopardize the main conclusions drawn from a genetic association study [24]. The *PDCD1* allele frequencies determined for the AML group were verified since there were no deviations from HWE in the examined data set.

### 3.3. Genetic Risk Associated with PDCD1 rs6710479

A Hardy–Weinberg equilibrium (HWE) calculator was used to examine the genetic risk associated with the PDCD1 rs6710479 mutation in allelic, dominant, codominant, and recessive genetic models [17]. The exploratory risk analysis was done with multiple univariate *t*-tests (Table 1, *p* value^1^). *PDCD1 rs6710479* associated with risk for AML, in the recessive model with odds ratio 1.6 (*p* = 0.013), in the dominant model at OR 1.5 (*p* = 0.038), in the allelic model (T vs. C) with OR 1.4 (*p* = 0.006), and in the codominant model at allele ratio 2:0 (TT vs. CC) with OR 1.94 (*p* = 0.005). To identify the best-fitting genetic model, a Bonferroni correction for multiple testing was applied (Table 1, *p* value^2^). The appropriate genetic models were allelic (HR 1.4, *p* = 0.04) and codominant at allele ratio 2:0 (HR 1.94, *p* = 0.04).

### 3.4. Clinical Characteristics According to PDCD1 rs6710479

**Figure 2:**
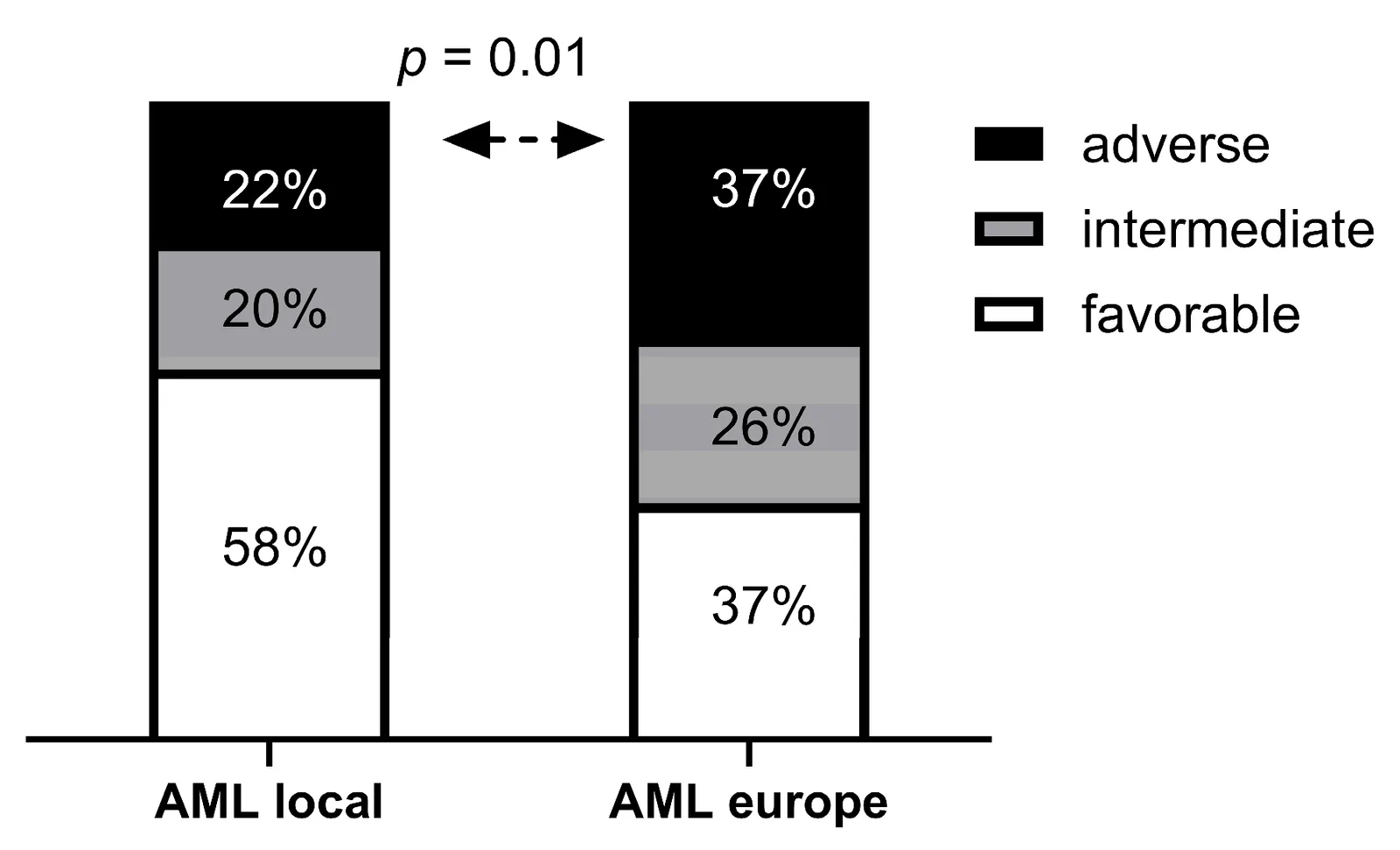
Bias in selection within the examined AML cohort. Variations in the ELN distribution within the local AML cohort versus an unbiased European AML cohort. The local AML group was chosen for a treatment involving consolidation therapy in first remission, with HDCT/ASCT.

The clinical features were examined in 139 AML patients diagnosed at a median age of 54 years, comprising 79 males and 60 females, resulting in a sex ratio (m/f) of 1.3. Baseline clinical attributes were examined for the whole cohort and for the three genetic subgroups of *PDCD1* rs6710479 composed of homozygous CC, heterozygous CT, or homozygous TT allele variants (Table 2). The median age at diagnosis was 53–54 years in CT and TT and 56 years in the CC subgroups (*p* = 0.68). Anemia was equally prevalent in all subgroups, with hemoglobin <100 g/L in 53–70% of patients and median hemoglobin levels of 82–90 g/L. Leukocyte counts (WBC) differed according to *PDCD1* genotype, with high counts in the TT, intermediate counts in the CT, and normal counts in the CC subgroups (WBC median 27, 15, and 7 g/L). Leukocytosis was more prevalent in the TT and CT subgroups (WBC > 10 g/L, 58% and 51% vs. 30%, *p* = 0.03). Platelet counts (PLT) differed according to *PDCD1* genotype, with low counts in the TT subgroup, intermediate counts in the CT subgroup, and high counts in the CC subgroup (PLT median 62, 80, 104 g/L, *p* = 0.07). Thrombocytopenia was more prevalent in the TT subgroup (PLT < 50 g/L, 50% vs. 27%, *p* = 0.07). LDH levels were equally high in the three subgroups (median 707 U/L). Peripheral blast percentage was elevated in the TT subgroup (median 56% vs. 44%, *p* = 0.29), with significantly higher bone marrow infiltration in the TT subgroup (median 90% vs. 60%, *p* = 0.02). The FAB classifications varied in the three genetic subgroups, with a prevalence of myeloblastic M2 and a lack of myelomonocytic M4 in the CC subgroup (46% and 9%). Cytogenetic anomalies and somatic mutations were observed at equal levels in all three genetic subgroups. The European LeukemiaNet (ELN) risk stratification categorized 79 (57%) cases as favorable, 28 (20%) as intermediate, and 31 (22%) as adverse risk, with equal ELN risk distribution in the three genetic subgroups. The ELN risk distribution within the local AML group, however, varied significantly (*p* = 0.001) from the ELN distribution in an unbiased AML cohort, which comprised 575 (37%) favorable, 410 (26%) intermediate, and 585 (37%) adverse risk cases [20] (Figure 2). The remission status prior to ASCT was predominantly CR (98%), with few MRD positive (6%) and very few PR (2%), with a similar distribution in the genetic subgroups. The number of infused stem cells varied, with a median transfusion of 3.7 × 10^6^/kg CD34+ PBSC (range 1–11 × 10^6^/kg).

### 3.5. Clinical Outcomes After HDCT/ASCT According to PDCD1 rs6710479

**Figure 3:**
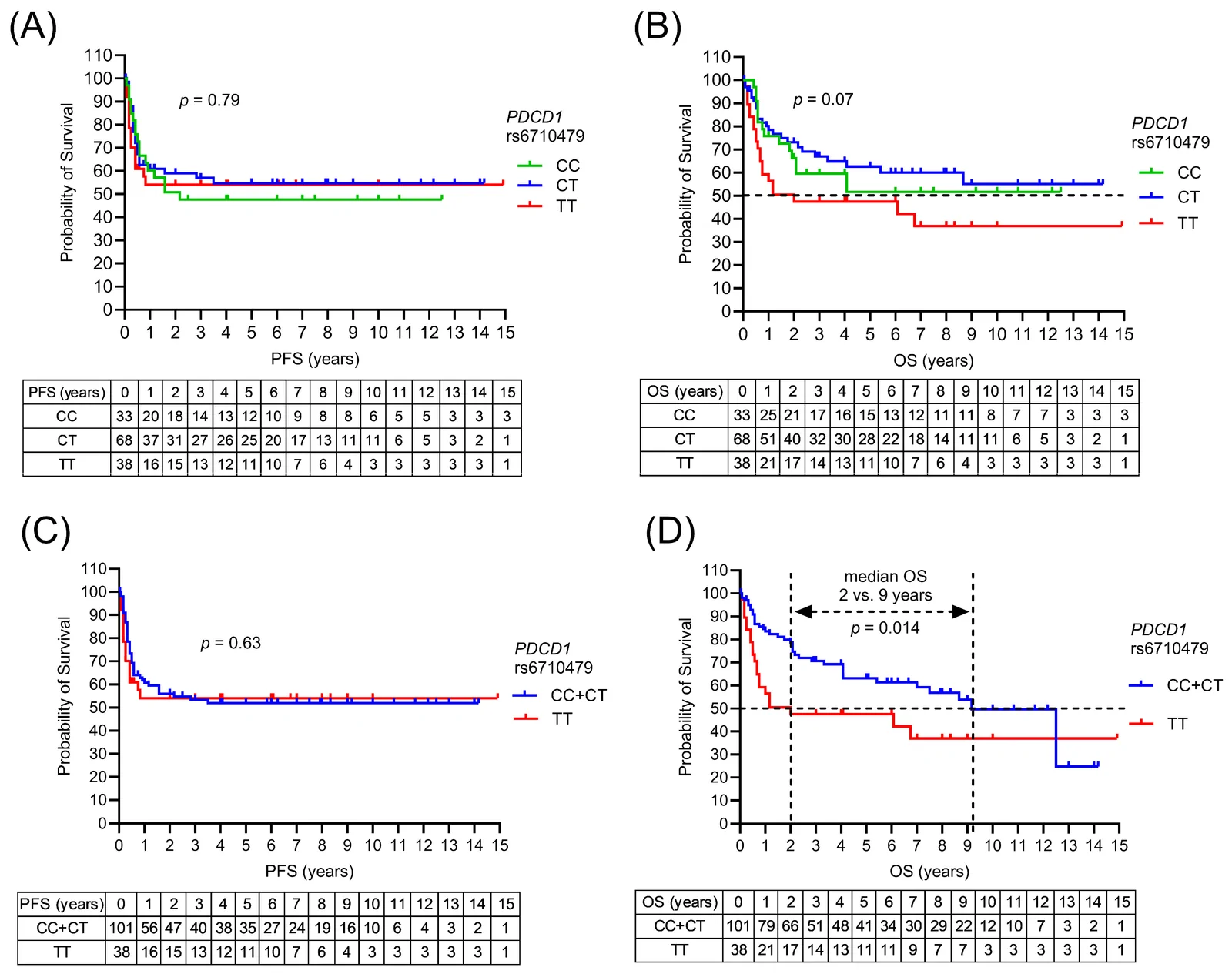
Survival outcomes after HDCT/ASCT according to *PDCD1* rs6710479 genotypes. Progression-free survival (**A**) and overall survival (**B**) according to *PDCD1* genotypes CC, CT, TT. Progression-free survival (**C**) and overall survival (**D**) according to *PDCD1* genotypes CC + CT versus TT.

The AML patients received standard induction therapy that included cytarabine and anthracycline, followed by consolidation therapy in the first remission with high-dose chemotherapy and autologous stem cell transplantation (HDCT/ASCT). Clinical outcomes were analyzed in the three *PDCD1* genetic subgroups (Figure 3A,B, Table 3). CR rates were very high at 92–96% in all three subgroups. Clinical outcomes differed according to *PDCD1* genotype, with one-year PFS rates of 59%, 56%, and 55% (*p* = 0.89) and one-year OS rates of 76%, 78%, and 53% (*p* = 0.018) in the CC, CT, and TT subgroups, respectively (Table 3). Relapse rates were similar in all subgroups, while death rates differed according to *PDCD1* genotype, with 24% versus 47% early death (within one year after HDCT/ASCT) in the CC and CT versus the TT subgroup (*p* = 0.024). In the combined analysis, median PFS was similar in all subgroups (Figure 3C), while median OS differed according to *PDCD1* genotype, with 2 years in the TT subgroup versus 9 years in the combined CC and CT subgroups (*p* = 0.014) (Figure 3D). Clinical outcomes in the TT subgroup were characterized by early disease progression and early death due to relapse-related mortality, in the CC and CT subgroups by early disease progression and prolonged OS. To identify independent prognostic factors, hazard ratios were calculated in a univariate and multivariate analysis (Table 4). While there was an increased risk of death associated with the *PDCD1* rs6710479 in the univariate analysis, this association was no longer evident in the multivariate analysis. Adverse ELN risk at diagnosis was an independent predictor for reduced OS in both univariate and multivariate analyses (HR 1.9 and 2.7). Age associated with elevated risk of death (HR 1.7 and 2.1). Leukocytosis was an independent predictor of reduced survival in multivariate analysis (HR 2, *p* = 0.04). Thrombocytopenia was an independent predictor of reduced survival (HR 0.6, *p* = 0.06). Peripheral blast cells > 50% were a predictor for disease progression in univariate analysis (HR 1.7, *p*= 0.05). Notably, in the multivariate analysis, *CTLA4* rs231775 T17hom associated with significant risk of disease progression and death (HR 2, *p* = 0.02), while there was no risk associated with the *PDCD1* rs6710479 (HR 1.1, *p* = 0.7) or the *LAG3* rs870849 variant (HR 0.9, *p* = 0.8). The clinical impact of the three immune checkpoint variants varies, with independent risk associated with the *CTLA4* variant, while the *PDCD1* and *LAG3* variants may primarily affect baseline disease characteristics with dependent associated effects on clinical outcomes.

## 4. Discussion

We examined the frequency of the common gene variant *PDCD1* rs6710479 in the genomes of AML patients in this retrospective single-center analysis, as well as the related gene risk in the development of AML, the associated clinical features, and the associated clinical outcomes. AML patients had higher allele frequencies of *PDCD1* rs6710479, with MAF 0.52 as opposed to MAF 0.43 in the European population. *PDCD1* rs6710479 was an AML risk gene in the allelic and codominant genetic models, with TT homozygote carriers having a twofold increased risk compared to CC homozygote carriers. *PDCD1* rs6710479 associated with clinical characteristics pertaining to blood count profiles: Leukocytosis was prevalent in the TT and CT subgroups, while thrombocytopenia prevailed in the TT subgroup. *PDCD1* rs6710479 may be a common risk allele for AML associated with specific clinical characteristics related to leukocyte and thrombocyte imbalances. These cellular aberrancies may be related to immune thrombocytopenia (ITP) entailing a complex immunological dysregulation, which can be modulated by germline variants of immune checkpoint regulators [25]. We previously studied the prevalence and impact of the common *CTLA4* variant rs231775 with associated ELN risk and treatment outcomes in the same AML cohort [13] and of the *LAG3* germline variant rs870849 with elevated allele frequency, associated disease risk, and associated leukocyte and thrombocyte imbalance [14]. Similar to *LAG3* rs870849, *PDCD1* rs6710479 revealed increased allele frequency, linked to disease risk, associated leukocytosis, and thrombocytopenia in the same AML cohort. LAG3 and PD-1 are co-inhibitory immune checkpoints that work through both overlapping and distinct pathways to regulate T cell activation and exhaustion. Therefore, dual blockade strategies targeting PD-1 and LAG3 have gained attention due to the significant potential to enhance anti-tumor immune responses [26]. CTLA4 blockade can enhance the graft-versus-leukemia effect in AML patients treated with an allogeneic transplant [27].

Various *PDCD1* genetic variants have been associated with an elevated cancer risk, including rs36084323, rs11568821, rs10204525, rs2227981, and rs2227982, with the latter two associated with treatment outcomes in AML with standard chemotherapy [28,29,30]. All *PDCD1* SNPs are located within a 9600 bp genomic region, with a high probability of inheritance in one unit (linkage block), including the variant rs6710479.

Overall, our findings are consistent with a paradigm that suggests that having at least one C allele—specifically, the homozygous CC genotype—provides a more favorable immunological and biological background in AML, both at diagnosis and after ASCT. Similar dynamics in the molecular mechanisms underlying both processes are indicated by the dose-dependent effects of *PDCD1* rs6710479 on AML disease progression and clinical feature modulation. The proper ratio of T-cell activity to T-cell exhaustion may be linked to the underlying molecular pathways [31,32,33]. The assertions that follow are based on hypotheses. *PDCD1* rs6710479 may lead to a lack of immune surveillance and an increase in fatigued T-cells in the pathophysiology of AML. *PDCD1* rs6710479 may suppress T-cell activity and increase the number of leukocytes and leukemic blast cells in AML disease. Furthermore, individual variations in post-ASCT outcomes in AML may be influenced by PD-1-mediated immune modulation. The possibility that disinhibited T cells may recapitulate a graft versus leukemic effect in the autologous situation is raised by the improved survival observed in CC and CT carriers. This is biologically plausible because endogenous tumor-reactive T cells have been shown to persist over time following HDCT/ASCT, correlate with clinical responses [34], and may be selectively favored in people with the hypomorphic rs6710479 variation. Future research is needed to determine the full complexity of the molecular pathways involving different *PDCD1* variations.

The primary limitations of the study include sample size, a retrospective single-site design, and selection bias, as most patients had favorable-risk disease. The investigation was exploratory because of the limited sample size, which heightened the likelihood of false positive and false negative results. Allele frequency and gene risk estimates may have been impacted by the selection bias, as most patients had favorable-risk disease. The allele frequency comparison was conducted using a large European database instead of a locally matched control group. Consequently, it was not feasible to differentiate between prognostic and predictive values. The results shown might indicate disease traits linked to genotypes but need careful interpretation and serve as ideas for future research. Sufficiently powered case–control studies will be necessary to formally assess the influence of *PDCD1* rs6710479 on AML susceptibility, its relationship with clinical features, and its effect on treatment results.

## 5. Conclusions

The prevalence and effects of the *PDCD1* immune checkpoint germline variant rs6710479 were investigated in this retrospective single-center study in AML patients eligible for HDCT/ASCT. *PDCD1* rs6710479 was linked to a dose-dependent rise in AML risk and a greater minor allele frequency in the local AML cohort when compared to the European reference population. Baseline blood count profiles varied among *PDCD1* genotypes, indicating an association of the germline variant *PDCD1* rs6710479 with leukocyte/platelet imbalance in AML disease. *PDCD1* rs6710479 may primarily affect baseline disease characteristics with dependent secondary effects on clinical outcomes.

## Figures and Tables

**Table 1:** Genetic risk associated with *PDCD1 rs6710479* in the local AML cohort.

Genetic Model	Comparison	OR (95% CI)	Z-Score	*p*-Value^1^	*p*-Value^2^
Recessive	CT vs. (TT + CC)	1.60 (1.10–2.33)	2.48	0.013	0.09
Dominant	(TT + CT) vs. CC	1.51 (1.02–2.23)	2.07	0.038	0.27
Allelic	T vs. C	1.40 (1.10–1.77)	2.78	0.006	0.04
Codominant	TT vs. CC	1.94 (1.22–3.09)	2.78	0.005	0.04
Codominant	TT vs. CT	1.44 (0.97–2.14)	1.80	0.071	0.50
Codominant	CT vs. CC	1.35 (0.89–2.04)	1.40	0.162	0.99

OR: odds ratio; CI: confidence interval. *p*-value^1^ without and *p*-value^2^ with Bonferroni correction for multiple testing (α = 0.05/7 = 0.00714).

**Table 2:** Baseline clinical characteristics according to *PDCD1* rs6710479 genotype.

Parameter	CC (*n* = 33)	CT (*n* = 68)	TT (*n* = 38)	All Patients (*n* = 139)	*p*
Male, *n* (%)	16 (57)	36 (57)	27 (55)	79 (56)	0.41
Age, median (range)	56 (19–74)	53 (20–73)	54 (17–70)	54 (17–74)	0.78
Hemoglobin g/L, median (range)	83 (43–123)	93 (7–137)	89 (47–130)	88 (7–137)	0.16
Anemia (Hb < 100 g/L), *n* (%)	25 (76)	45 (66)	27 (71)	97 (70)	0.61
Leukocytes g/L, median (range)	6 (1–214)	11 (1–272)	21 (1–202)	11 (1–272)	0.08
Leukopenia (WBC < 4 g/L), *n* (%)	12 (36)	17 (25)	9 (24)	38 (22)	0.43
Leukocytosis (WBC > 10 g/L), *n* (%)	12 (36)	34 (50)	26 (68)	72 (52)	0.03
Hyperleukocytosis (WBC > 100 g/L), *n* (%)	1 (3)	7 (10)	5 (13)	13 (9)	0.32
Platelets g/L, median (range)	104 (6–714)	80 (8–184)	62 (4–229)	83 (4–714)	0.07
Thrombocytopenia (PLT < 50 g/L), *n* (%)	9 (27)	21 (31)	19 (50)	48 (35)	0.07
LDH U/L, median (range)	695 (141–7108)	725 (116–4887)	700 (152–2737)	707 (116–7108)	0.96
LDH > 1000 U/L, *n* (%)	11 (33)	15 (22)	9 (24)	35 (25)	0.46
Blast cells PB %, median (range)	44 (0–98)	45 (0–99)	56 (5–99)	46 (0–99)	0.29
Blast cells BM %, median (range)	60 (15–95)	80 (0–100)	90 (11–95)	80 (0–100)	0.02
FAB classification, *n* (%)					0.25
M0	4 (12)	5 (7)	2 (5)	11 (8)	0.85
M1 *	8 (24)	20 (29)	8 (21)	36 (26)	0.62
M2	15 (46)	18 (26)	11 (29)	44 (32)	0.14
M4	3 (9)	18 (26)	11 (29)	32 (23)	0.09
M5	2 (6)	5 (7)	4 (10)	11 (8)	0.78
Pathogenesis					0.18
de novo AML, *n* (%)	29 (91)	61 (94)	32 (86)	122 (91)	
sAML (MDS/MPN-related), *n* (%)	0	3 (5)	3 (8)	6 (4)	
tAML (therapy-related), n (%)	3 (9)	1 (2)	2 (5)	6 (4)	
Cytogenetic aberrations, *n* (%)					0.15
Normal karyotype	19 (59)	48 (73)	20 (57)	87 (65)	
Abnormal karyotype ^1^	12 (38)	18 (27)	15 (43)	45 (34)	
Complex karyotype	1 (3)	0	0	1 (1)	
Somatic mutations, *n* (%)					
normal	5 (18)	7 (11)	8 (24)	20 (16)	0.23
NPM1_mut_ FLT3_wt_ (favorable)	8 (29)	20 (30)	9 (26)	37 (29)	0.92
FLT3-ITD NPM1_wt_ (intermediate)	1 (4)	3 (5)	3 (9)	7 (5)	0.59
FLT3-ITD NPM1_mut_ (intermediate)	3 (11)	12 (18)	3 (9)	18 (14)	0.42
Adverse risk genes ^2^	7 (25)	13 (20)	6 (18)	26 (20)	0.76
Intermediate risk genes ^3^	13 (46)	30 (44)	14 (41)	57 (45)	0.90
ELN-risk classification (2022), *n* (%)					0.83
Favorable (ELN1)	21 (58)	36 (52)	23 (60)	80 (58)	
Intermediate (ELN2)	6 (21)	16 (24)	6 (16)	28 (20)	
Adverse (ELN3)	6 (21)	16 (24)	9 (24)	31 (22)	
Remission status before HDCT/ASCT					0.46
CR CR, MRD positive PR	32 (97) 2 (6) 1 (3)	67 (98) 5 (7) 1 (2)	36 (95) 2 (5) 2 (5)	135 (97) 9 (6) 4 (2)	
CD34+ SC infused, *n* × 10^6^/kg	3.9 (1.5–11)	4.0 (1–7.3)	3.4 (1–10.8)	3.7 (1–11)	0.39

* including AML not further classifiable, AML from MDS (WHO 2008), and AML from MPN (WHO 2008). ^1^ including t(8;21), inv16, t(9;22)(q34.1;q11.2), t(6;9)(p23.3;q34.1), t(v;11q23.3), t(8;16)(p11.2; p13.3), inv(3)(q21.3q26.2), t(3;3)(q21.3;q26.2), t(3q26.2;v), −5, or del(5q); −7; −17/abn(17p). ^2^ including *ASXL1*, *BCOR*, *EZH2*, *RUNX1*, *SF3B1*, *SRFS2*, *STAG2*, *U2AF1*, *TP53*, *ZRSR2*. ^3^ including *DNMT3A*, *TET2*, *IDH1*, *IDH2*, *KRAS*, *NRAS*, *KIT*. WBC: white blood cell count; PLT: platelets; PB: peripheral blood; BM: bone marrow; ELN: European LeukemiaNet; FAB: French–American–British; LDH: lactate dehydrogenase; MDS: myelodysplastic syndrome; MPN: myeloproliferative neoplasm; mut: mutated; wt: wild type; HDCT: high-dose chemotherapy; ASCT: autologous stem cell transplant; CR: complete remission; MRD: minimal residual disease; PR: partial remission; SC: stem cells.

**Table 3:** Clinical outcomes and survival according to *PDCD1* genotype, univariate analysis.

Outcomes and Survival	CC (*n* = 33)	CT (*n* = 68)	TT (*n* = 38)	All Patients (*n* = 139)	*p*
CR, *n* (%)	31 (94)	65 (96)	35 (92)	134 (96)	0.72
Relapse, *n* (%)	16 (48)	28 (41)	16 (42)	60 (43)	0.90
One-year PFS, *n* (%)	20 (61)	40 (59)	21 (55)	80 (56)	0.89
Death, *n* (%)	16 (48)	24 (35)	21 (55)	68 (49)	0.13
Early death ^1^, *n* (%)	8 (24)	17 (25)	18 (47)	31 (22)	0.024
One-year OS, *n* (%)	25 (76)	53 (78)	20 (53)	97 (70)	0.018
Two-year OS, *n* (%)	20 (61)	48 (70)	18 (47)	86 (62)	0.06

CR: complete remission; OS: overall survival; PFS: progression-free survival. ^1^ within one year after ASCT.

**Table 4:** Predictors in clinical outcomes and univariate and multivariate analysis.

	Univariate				Multivariate			
Predictors	PFS		OS		PFS		OS	
	HR (CI)	*p*	HR (CI)	*p*	HR (CI)	*p*	HR (CI)	*p*
*PDCD1* rs6710479, TT *CTLA4* rs231775, T17hom *LAG3* rs870849, I455hom	1.2 (0.7–2.1) 1.8 (1.1–3.0) 1.1 (0.6–2.1)	0.6 0.02 0.8	1.7 (1.1–2.9) 1.5 (0.9–2.5) 1.3 (0.7–2.4)	0.05 0.11 0.4	1.2 (0.6–2.2) 2.0 (1.1–3.5) 0.8 (0.4–1.6)	0.7 0.02 0.5	1.1 (0.6–2.1) 2.0 (1.1–3.7) 0.9 (0.4–1.9)	0.7 0.02 0.7
ELN risk, adverse	1.6 (0.9–2.8)	0.12	1.9 (1.1–3.3)	0.02	1.6 (0.8–3.1)	0.2	2.7 (1.4–5.3)	0.005
Age at ASCT, >65	0.7 (0.3–1.5)	0.3	1.7 (0.9–3.2)	0.1	0.7 (0.3–1.8)	0.5	2.1 (1.1–4.1)	0.06
WBC, >10 g/L PLT, >50 g/L Peripheral blast cells, >50%	1.6 (0.9–2.8) 0.8 (0.5–1.4) 1.7 (1.0–3.0)	0.1 0.4 0.05	1.4 (0.8–2.4) 0.7 (0.4–1.2) 1.3 (0.8–2.2)	0.2 0.2 0.3	1.5 (0.8–2.8) 0.8 (0.5–1.4) 1.5 (0.8–2.6)	0.2 0.4 0.2	2.0 (1.1–3.8) 0.6 (0.3–1.0) 1.0 (0.5–1.9)	0.04 0.06 0.9

ELN: European LeukemiaNet. WBC: white blood cell count. PLT: platelets.

## Data Availability

Data available on request due to restrictions, privacy, and ethics.

## Abbreviations

Acute myeloid leukemia (AML), lymphocyte-activation gene 3 (LAG3), cytotoxic T-lymphocyte associated protein 4 (CTLA4), programmed cell death protein 1 (PD-1), high-dose chemotherapy (HDCT), autologous stem cell transplant (ASCT), minor allele frequency (MAF).
